# Analysis of the Current Situation and Influencing Factors of Nurses' Voice Behavior in Neonatal Intensive Care Units of Grade A Tertiary Hospitals in Sichuan Province: A Multicenter Cross-Sectional Study

**DOI:** 10.1155/jonm/8175652

**Published:** 2025-03-24

**Authors:** Xiujuan Zhang, Qiong Chen, Yanling Hu, Xiufang Zhao, Xi Huang

**Affiliations:** ^1^Department of Neonatology Nursing, West China Second University Hospital, Sichuan University, Chengdu, China; ^2^Key Laboratory of Birth Defects and Related Diseases of Women and Children, Sichuan University, Ministry of Education, Chengdu, China; ^3^Department of Nursing, West China Second University Hospital, Sichuan University, Chengdu, China

**Keywords:** influencing factors, neonatal intensive care unit, nurses, Sichuan province, tertiary hospitals, voice behavior

## Abstract

**Aims:** Voice behavior refers to nurses' proactive actions in offering constructive suggestions, providing feedback, or raising concerns in the workplace, which are crucial for enhancing care quality and improving the work environment. This study aims to investigate the current status and influencing factors of voice behavior among nurses in neonatal intensive care units (NICUs) in tertiary hospitals in Sichuan Province, providing empirical evidence for improved nursing management and hospital administration.

**Design:** A multicenter, crosssectional survey.

**Methods:** From January to June 2023, 422 neonatal nurses from tertiary hospitals in Sichuan Province were selected through stratified random sampling. Data were collected through self-reported questionnaires, including a general information questionnaire and a voice behavior scale. The voice behavior scale consists of 10 items, divided into promotive and prohibitive behavior dimensions, using a five-point Likert scale (1 = “*never*” and 5 = “*always*”). The scale has been widely used among Chinese nurses and demonstrates good internal consistency (Cronbach's α = 0.951). Data analysis was conducted using SPSS Version 26.0. Structural validity was assessed through exploratory factor analysis (KMO > 0.8, Bartlett's test *p* < 0.05), followed by confirmatory factor analysis using AMOS. For group comparisons, independent *t*-tests and analysis of variance (ANOVA) were used, with Welch's test for unequal variances. Post hoc multiple comparisons were performed using Tamhane's T2 for unequal variances and LSD for equal variances. A *p*-value < 0.05 was considered statistically significant.

**Results:** Age, marital status, and number of children significantly influenced voice behavior. Voice behavior increased with age up to 50 years, unmarried individuals exhibited less voice behavior than married or divorced ones, and more children correlated with more voice behavior. Job title, position, and years of experience in the neonatal department also significantly impacted voice behavior. Higher positions and more than 15 years of experience were associated with increased voice behavior. Senior titles correlated with higher prohibitive voice behavior.

**Conclusion:** The voice behavior of NICU nurses is influenced by various factors, including age, marital status, number of children, job title, position, and years of experience in the neonatal department. As age increases, the number of children grows, work experience accumulates, and nurses' voice behavior tends to intensify. In particular, for senior nurses, managers should pay attention to their prohibitive voice behavior and encourage their active involvement in decision-making processes to enhance the quality of care. Nursing managers should tailor management strategies based on these individual characteristics, providing customized support for nurses at different experience levels. At the same time, emphasis should be placed on creating a psychologically safe work environment to stimulate nurses' initiative and creativity, thereby improving team communication and collaboration. This approach will contribute to ensure the quality of care and patient safety in NICUs.

**Implications for the Profession:** Understanding the factors influencing voice behavior helps nursing managers to enhance nurse participation and care quality. Nursing managers can implement the following strategies: (1) create a psychologically safe environment: encourage open communication by ensuring nurses feel their opinions are valued, with clear channels for feedback and action, (2) address senior nurses' prohibitive voice behavior: provide leadership training to senior nurses to transform negative feedback into constructive suggestions, promoting collaboration and work improvement, (3) tailor strategies based on experience: offer support and mentorship to new nurses, while encouraging experienced nurses to take leadership roles and contribute to decision-making, (4) incentivize contributions: develop reward systems to recognize nurses' involvement in improving patient care, such as acknowledging innovative ideas and active participation.

## 1. Introduction

The neonatal intensive care unit (NICU) primarily admits critically ill newborns within 28 days of birth, who are often at risk of life-threatening conditions or already suffering from severe diseases. Due to the rapid changes in their condition and high mortality rates, continuous and close monitoring by healthcare staff is required. NICU nurses play a crucial role in this process. However, due to the heavy workload, frequent emergencies, and close team collaboration, NICU nurses experience significantly higher levels of occupational stress compared to nurses in general pediatrics or adult wards, which can lead to burnout, mental health issues, and a decline in the quality of care [1–3]. Additionally, a high-pressure work environment may increase the risk of social media addiction, which in turn can affect nurses' work performance and quality of life, and it is also associated with an increased risk of medical safety incidents and occupational injuries among nurses [3, 4].

In today's high-pressure medical environment, the proactive voice behavior of nurses is particularly important. Since the concept of nurse voice behavior was introduced in 2015, it has been widely recognized as the proactive behavior of nurses in offering suggestions and opinions to improve the organization. This behavior reflects the individual's sense of ownership and emotional expression, significantly promoting organizational development and solving practical problems [5, 6]. Voice behavior can be divided into promotive voice and prohibitive voice. Promotive voice refers to employees offering innovative ideas or suggestions aimed at improving the work environment or enhancing organizational performance, while prohibitive voice mainly focuses on identifying problems or potential negative factors in the work process, with the goal of avoiding or reducing the negative impact of these factors on the work [7]. Studies show that the voice behavior of nonmanagement nurses not only enhances their sense of participation and decision-making authority but also improves job satisfaction, reduces stress, and thus improves mental health [8, 9]. Hospitals that encourage nurses to participate in decision-making and voice their opinions typically have higher levels of job satisfaction and mental health among their nurses than those that do not, and the nurses in these hospitals also report lower work stress [9]. Additionally, involving nurses in nursing management helps them better understand and implement work processes, reducing resistance and uncertainty in decision-making. For example, regular meetings involving nurses in work improvement can allow nurses to make specific suggestions about nursing quality and workflow optimization, helping hospitals to identify and resolve practical issues in a timely manner. This participation not only enhances the quality of care but also significantly reduces nurse turnover and professional burnout [10]. By encouraging nurse voice behavior, hospitals can optimize communication channels, improve the work environment, and thus enhance overall work efficiency and patient satisfaction. For individuals, voice behavior can increase a sense of professional achievement, alleviate stress, and promote harmonious work relationships; for teams, it helps to create a positive work atmosphere and culture, drive departmental progress, and assist in identifying risks that leaders may not notice [11, 12].

In China, voice behavior has been valued since ancient times. Confucius once said, “Honest advice may be unpleasant, but it benefits actions,” and during the feudal era, institutions such as the Imperial Court of Censors were established, reflecting the cultural tradition of offering advice. With the rapid development of the global socioeconomic landscape and changes in organizational environments, the significance and scope of voice behavior have been continuously emphasized. Internationally, the concept of voice behavior originated from the EVI model proposed by American economist Hirschman [13]. Later, scholars such as Rusbult et al. [14] and LePine and Van Dyne [15] further explored the motivations and manifestations of individual voice behavior, suggesting that voice behavior is not only an active response driven by emotional support but can also go beyond job responsibilities to foster organizational improvement.

In the field of nursing, nurses are often the first to identify potential problems within the healthcare organization, and silence can lead to serious medical safety incidents. In neonatal care, where the work is complex and involves vulnerable newborns, nurses' voice behavior is particularly important. Although there have been numerous studies exploring the influencing factors and outcomes of voice behavior in other fields, research on the voice behavior of nurses, particularly those in neonatal departments, remains relatively scarce. The current literature primarily examines the impact of factors such as work pressure and job satisfaction on voice behavior, but there is little research that specifically analyzes the voice motivation and behavior characteristics of neonatal nurses in high-risk environments. This study aims to fill this research gap by investigating the current status and influencing factors of neonatal nurses' voice behavior based on data from 422 neonatal nurses in tertiary hospitals in Sichuan Province. The study seeks to provide strategic guidance for hospital management, encourage nurses to actively voice their opinions, and ultimately improve the quality of medical services and reduce potential risks.

## 2. Materials and Methods

### 2.1. Study Design

This multicenter cross-sectional study was conducted from January to September 2023 in the NICUs of Grade A tertiary hospitals in Sichuan Province, China. The study received approval from the relevant ethics committees (Medical Research 2023, Ethics Review No. [042]). It was carried out in strict accordance with the requirements of the World Medical Association's Declaration of Helsinki. All participants were fully informed and provided electronic consent prior to the commencement of the research. The study adhered to ethical principles of confidentiality and nonmaleficence to protect the rights of the participants.

### 2.2. Participants Recruitment and Eligibilities

Inclusion criteria were as follows: registered and currently practicing nurses, no physical or psychological illnesses that could affect clinical work during the survey period, no major life events (such as divorce, death of a loved one, severe illness, serious accidental injury, or significant financial loss) in the past 6 months, and voluntary participation in this study.

Exclusion criteria included the following: nurses currently undergoing standardized training or department rotation, nurses in advanced studies, probationary nurses, retired or rehired nurses, and those who had previously participated in similar surveys. Exclusion criterion was a questionnaire completion time of less than 2 min.

The sample size was calculated according to the standard of 5–15 times the number of questionnaire items, with an additional 10%–20% added for nonresponse rate [16]. The questionnaire contains 10 items. Using the middle multiplier of 10, the required sample size for this study was determined to be 100 participants. Considering a 20% nonresponse rate, the final minimum sample size for this study was determined to be 120 participants.

This study adopted a stratified cluster sampling method to select nurses working in NICUs of tertiary public medical institutions in Sichuan Province from January to September 2023 as research subjects. The sample selection followed these steps:

1. Target hospital screening: From 2534 medical institutions registered with the Sichuan Provincial Health Commission, 120 tertiary nonprofit public hospitals were selected. Through official websites, 77 institutions with neonatal units were identified, among which 54 had independent neonatal departments. After verification through the Sichuan Neonatal Collaboration Platform, two hospitals without neonatal departments were excluded, resulting in 52 eligible target institutions.
2. Sample stratification and questionnaire distribution: Data stratification was conducted based on the proportion of nurses in each region. Electronic questionnaires were distributed through hospital liaisons, with data updated every 2 weeks to ensure that the sample size in each region accounted for at least 10% of the total number of nurses, thereby ensuring regional representativeness. For regions with fewer than 10 nurses, if representative samples were not obtained in the first round of data collection, no further data collection was conducted.
3. Formal investigation in two rounds:
4. First round: Questionnaires were distributed to 52 hospitals through the Sichuan Neonatal Collaboration Platform, and 432 questionnaires were collected. The following invalid questionnaires were excluded ①from nontertiary public hospitals (17), ②from participants with physical/psychological conditions potentially affecting clinical work (13), ③from participants experiencing major life events in the past 6 months (42), and ④questionnaires completed in less than 2 min (33).
5. A total of 327 valid questionnaires were obtained. Regional data analysis revealed insufficient responses in some regions (e.g., Dazhou, Guangyuan, Liangshan, Luzhou, and Ya'an), prompting additional data collection in communication with hospital liaisons.
6. Second round: Additional data collection was conducted in 31 target hospitals, resulting in 143 questionnaires. The following invalid questionnaires were excluded ①from nontertiary public hospitals (20), ②from participants with physical/psychological conditions potentially affecting clinical work (21), ③from participants experiencing major life events in the past 6 months (58), ④questionnaires completed in less than 2 min (49), and ⑤from probationary nurses (4) and retired nurses (1).
7. Ultimately, a total of 422 valid questionnaires were obtained across both rounds, with an efficiency rate of 73.39%.

### 2.3. Measurement Tools

#### 2.3.1. General Information Questionnaire

The questionnaire, designed by the researchers, includes the following items: gender, age, ethnicity, only-child status, personal health status, marital status, number of children, location of the workplace, nature of the workplace, educational background, job title, position, years of experience in neonatal care, and any significant family or personal events in the past 6 months.

#### 2.3.2. Voice Behavior Scale

The questionnaire, developed by Liang, Farh, and Farh [17] in the context of Chinese enterprises, has been widely used among nurse groups and public sector employees in China. It is currently one of the most frequently used and locally adapted research tool in the field of organizational behavior studies, with a Cronbach's α coefficient ranging from 0.84 to 0.90. The questionnaire consists of 10 items, divided into two dimensions: promotive behavior (5 items) and prohibitive behavior (5 items). It uses a Likert five-point scale, where 1 represents “*never*” and 5 represents “*always*,” with a total score of 50. Higher scores indicate a higher frequency of voice behavior. In this study, the overall Cronbach's α coefficient for the questionnaire is 0.951, with Cronbach's α values for promotive voice and prohibitive voice being 0.950 and 0.911, respectively.

### 2.4. Survey Methods and Quality Control

#### 2.4.1. Questionnaire Design

Based on the research objectives and literature review, a questionnaire containing both multiple-choice and rating questions was designed. The content of the questionnaire mainly covers the following two aspects: (1) general information survey: Demographic information such as age, gender, ethnicity, and years of work experience. (2) Voice behavior scale: This includes five items on promotive behavior and five items on prohibitive behavior. Promotive behavior: A1: I think about and propose my own suggestions for potential issues in the department. A2: I actively propose new plans and projects that would benefit the department. A3: I actively suggest improvements to the department's workflow. A4: I proactively offer rational suggestions to help the department achieve its goals. A5: I propose constructive ideas to improve the department's operations. Prohibitive behavior: A6: I promptly discourage other employees' behaviors that affect work efficiency in the department. A7: I speak the truth about serious issues that may cause losses to the department, even if others have different opinions. A8: I actively express my views on phenomena that affect work efficiency in the department, even if it might embarrass others. A9: When problems arise in the department's work, I dare to point them out without worrying about offending others. A10: I actively report issues that need coordination in the work to the department leadership.

#### 2.4.2. Pilot Study

Before the formal survey, a pilot test of the questionnaire was conducted. The sample size for the pilot study should be 20%–30% of the total sample size [18]. Given the calculated sample size of 120, the pilot study involved 24–36 participants. Using a convenience sampling method, 30 NICU nurses from Chengdu were selected for the pilot test. The pilot test assessed the clarity and validity of the questionnaire, and feedback was used to make necessary modifications and adjustments, ensuring that the questionnaire was accurate and easy to understand. Reliability testing showed a Cronbach's α coefficient of 0.966 for the voice behavior scale, indicating high internal consistency. Exploratory factor analysis (EFA) results showed a Kaiser–Meyer–Olkin (KMO) coefficient of 0.938 for the voice behavior scale, and Bartlett's test of sphericity was significant at < 0.001, indicating that the data were suitable for factor analysis.

#### 2.4.3. Questionnaire Distribution

For the formal survey, a stratified cluster sampling method was used to study the target population in hospitals that met the inclusion and exclusion criteria. The survey was distributed via the JINSHUJU platform (https://jinshuju.net/signin), an efficient, convenient, secure, and highly anonymous online questionnaire and data collection tool. The questionnaire link generated by the platform was distributed to NICU nurses in tertiary public medical institutions in Sichuan Province through email and social media (e.g., WeChat).

#### 2.4.4. Data Collection

Respondents completed the questionnaire online by clicking on the provided link. Before filling out the questionnaire, an electronic informed consent form was presented to the participants via a QR code. This form explained the purpose, content, and requirements of the study in detail. Participants could only start the survey after selecting “Accept.” The questionnaire included standardized instructions, with all items being mandatory, and responses were submitted anonymously to ensure participant privacy. The JINSHUJU platform automatically collected and stored the questionnaire data, providing preliminary statistics and organization. During data collection, the platform's backend management features allowed for real-time monitoring of questionnaire completion progress and data quality, ensuring the completeness and validity of the collected data.

#### 2.4.5. Data Export and Analysis

Upon completion of data collection, all questionnaire data were exported from the JINSHUJU platform in Excel format. The exported data included all respondents' answers, along with detailed response times and completion durations for each question. After exporting the data, it was cleaned and organized, with invalid responses and clearly unreasonable data removed.

The study involved 52 eligible tertiary hospitals with neonatal units in Sichuan Province. A total of 575 questionnaires were collected, achieving a 100% response rate. After downloading the data, the researchers checked the completeness and validity of the questionnaires, excluding those that did not meet the criteria. Ultimately, 422 valid questionnaires were obtained, with an effective rate of 73.39%.

### 2.5. Statistical Analysis

This study used SPSS Version 26.0 software for data analysis. For continuous variables with a normal distribution, descriptive statistics were presented using ($\overset{¯}{\chi }\pm s$); for categorical variables, frequency and percentage were used. The internal consistency and construct validity of all measurement models were examined. First, EFA was conducted to assess construct validity, with the criteria of KMO value > 0.8 and Bartlett's test of sphericity with a *p*-value < 0.05, indicating that the data were suitable for factor analysis. Subsequently, confirmatory factor analysis (CFA) was performed for further testing, with the following criteria: CMIN/DF < 5, RMSEA < 0.1, TLI > 0.9, CFI > 0.9, NFI > 0.9, and SRMR < 0.05. These standards indicate a good model fit. For between-group comparisons, independent sample *t*-tests or one-way analysis of variance (ANOVA) were used for continuous variables with normal distribution. When the assumption of homogeneity of variance was not met, Welch's test was used. For variables with significant differences, post hoc multiple comparisons were performed. Specifically, when the homogeneity of variance assumption held, least significant difference (LSD) was used, while Tamhane's T2 test was applied when the assumption did not hold, ensuring robust and reliable results for multiple comparisons. To effectively control the cumulative risk of Type I errors, Bonferroni correction was applied in the LSD multiple comparisons, adjusting the significance level to *α*/*k*, where *k* represents the number of comparisons. Consequently, for the LSD multiple comparisons, statistical significance was determined based on the corrected threshold, while for other analyses, the standard significance level of *p* < 0.05 was used. All results reported as significant met the respective thresholds, ensuring the reliability of the findings.

## 3. Results

### 3.1. General Characteristics of Participants

Among the 422 nurses surveyed, 99.3% were female. The age distribution was mainly concentrated between 30 and 39 years (52.8%), followed by 30 years and below (32.0%). The majority were of Han ethnicity (96.0%), followed by Tibetan (3.1%) and Yi (0.9%) ethnicities. Only 31.3% were only children. Regarding marital status, 75.6% were married, and 21.8% were unmarried. Additionally, 31.3% of the nurses had no children, while 46.7% had one child. Most of the participants held a bachelor's degree (82.0%), with only 2.6% having a master's degree or higher (Table 1). The study subjects were distributed across 16 prefecture-level cities and 2 autonomous prefectures in Sichuan Province, with the highest percentage of nurses from Chengdu (31.3%), followed by Nanchong (10.0%) and Mianyang (8.8%).

### 3.2. Other Work-Related Information

The study also collected information on the nature of the participants' workplaces, their professional titles, and positions. The survey revealed that 76.1% of the nurses worked in general hospitals, while 23.9% worked in specialized hospitals. The professional titles were primarily nurse practitioners and nurse supervisors, accounting for 45.7% and 41.5%, respectively, with only 5.2% holding senior titles. Most of the nurses were frontline staff, making up 79.4% of the participants. In terms of work experience, the majority had 0–5 years (36.7%) and 6–10 years (30.6%) of experience (Table 2).

### 3.3. Reliability Analysis of the Scale

The Cronbach's α for the total voice behavior scale was 0.951, with the Cronbach's α values for promotive voice and prohibitive voice being 0.950 and 0.911, respectively, indicating that the scale has good reliability (Table 3).

### 3.4. Common Method Bias Test

Given that the data were self-reported by the nurses, a single-factor CFA was used to test for the common method bias. The fit indices for the CFA model M1 were as follows: CMIN/DF = 4.146, RMSEA = 0.087, TLI = 0.967, CFI = 0.980, and SRMR = 0.000, indicating good model fit. The fit indices for the single-factor model M2 were as follows: CMIN/DF = 8.573, RMSEA = 0.134, TLI = 0.641, CFI = 0.654, and SRMR = 0.060, indicating poor model fit. The significant differences in fit indices between the two models suggest that the measurement items do not belong to a single factor, indicating that there is no significant common method bias in this study.

### 3.5. Voice Behavior Scores of NICU Nurses With Different Demographic Characteristics

The study results show significant differences in voice behavior based on age, marital status, and the number of children (*p* < 0.05). These variables also exhibited significant differences in both promotive and prohibitive voice behaviors (*p* < 0.05) (Table 4). Specifically, before the age of 50, voice behavior increased with age. Unmarried individuals demonstrated less voice behavior compared to married or divorced individuals. The more children the participants had, the more voice behavior they exhibited.

Furthermore, significant differences in voice behavior were observed based on job title, position, and years of experience in the neonatal department (*p* < 0.05) (Table 5). Specifically, higher positions were associated with more voice behavior. Nurses with more than 15 years of experience exhibited higher voice behavior compared to those with 6–15 years and 0–5 years of experience. Among those with junior titles and above, voice behavior increased with higher job titles. The differences in promotive and prohibitive voice behaviors were only evident in those with senior titles, and senior nurses exhibited significantly higher prohibitive voice behavior compared to other titles.

Using Tamhane's T2 for post hoc multiple comparisons, it was found that voice behavior before the age of 50 years increased with age. This distinction was present in both promotive and prohibitive voice behaviors. LSD was used for post hoc multiple comparisons of marital status and number of children. It was found that unmarried individuals exhibited less voice behavior (including both promotive and prohibitive) compared to married or divorced individuals. Married individuals showed significant differences from divorced individuals only in prohibitive voice behavior, while differences in promotive voice behavior were not significant (*p* > 0.05). Regardless of whether it was promotive or prohibitive voice behavior, the more children the participants had, the more voice behavior they exhibited, even after the Bonferroni adjustment (Table 6).

Post hoc multiple comparisons showed that higher positions were associated with more voice behavior. Nurses with more than 15 years of experience exhibited higher voice behavior compared to those with 6–15 years of experience, and those with 6–15 years of experience exhibited higher voice behavior compared to those with 0–5 years of experience. Among those with junior titles and above, voice behavior increased with higher job titles. Differences in promotive and prohibitive voice behaviors were mainly observed among associate chief nurses, with associate chief nurses exhibiting significantly higher prohibitive voice behavior compared to other titles. However, in terms of promotive voice behavior, there was no statistically significant difference between associate chief nurses and nurse supervisors.

## 4. Discussion

### 4.1. Analysis of Voice Behavior Scores Among NICU Nurses

The results of this study show that the voice behavior score of NICU nurses was (41.52 ± 7.11). According to Xiao et al.'s [19] classification based on latent profile analysis of voice behavior, this indicates that the voice behavior of NICU nurses in tertiary public hospitals in Sichuan Province falls under the balanced risk type. This type of individual typically considers the necessity and potential risks of voice behavior, making decisions after weighing the pros and cons, which aligns with the characteristics of voice behavior in most populations.

In this study, the promotive voice score was 21.26 ± 3.72, while the prohibitive voice score was 20.26 ± 3.83, with a statistically significant difference between the two (*p* < 0.001), suggesting that NICU nurses have deficiencies in prohibitive voice behaviors. Prohibitive voice typically manifests as warnings regarding existing work processes or policies, focusing on potential risks and problems, and may challenge existing leadership authority and systems. Relevant research indicates that [20] the low score for prohibitive voice often reflects the suppression of such behaviors within organizational culture, particularly in medical environments that emphasize stability and adherence to strict procedures. In these environments, employees may suppress their willingness to raise prohibitive voices due to respect for authority and fear of uncertainty. However, prohibitive voice is not entirely equivalent to dissatisfaction with the status quo; it can also be seen as an early warning mechanism, helping managers identify potential risks and shortcomings. Studies have shown that [21], especially in the healthcare field, prohibitive voice can provide feedback to management, helping to improve work processes and patient safety. Although prohibitive voice may be viewed as a challenge to leadership authority, in modern management philosophies, it is also considered an important part of organizational change, fostering bidirectional communication between employees and management, thereby promoting the long-term development of the organization [22]. Some studies further suggest that [23] the frequency of prohibitive voice is closely related to organizational culture and leadership styles. For example, in environments where the organizational culture supports innovation and open communication, employees such as nurses are more likely to raise prohibitive voice. Therefore, when the score for prohibitive voice is too low, it is necessary to cultivate an open communication culture and encourage employees to offer opinions and suggestions in order to improve this situation, ultimately enhancing the overall quality and efficiency of nursing services.

### 4.2. Analysis of Differences in Voice Behavior Across Different Demographic Characteristics

#### 4.2.1. Age

Many scholars suggest that with age, employees are more likely to engage in voice behavior. With accumulated work experience, older employees are better able to engage in critical thinking, identify problems and weaknesses in their work, and express their opinions and suggestions at more appropriate times. Additionally, as they age, employees tend to make more mature evaluations of risks and benefits, which allows them to speak up more strategically, rather than remaining silent due to the fear of expressing themselves or potential risks [24]. However, in this study, there was no significant difference in the voice behavior scores between nurses aged 50 years and above (43.33 ± 8.19) and those under 50 years (46.14 ± 5.28) in the NICU. This finding contradicts some previous studies, which suggest that older employees are more inclined to express their opinions as they age [25]. However, other research indicates that older employees may, in some cases, be more inclined to remain silent, especially in environments with more authority or hierarchical structures. Older employees may be more cautious about expressing their opinions due to long-established power dynamics or a preference for compliance [26]. In the case of NICU nurses, the lack of a significant difference between nurses aged 50 years and above and those below 50 years may be related to the unique work environment and leadership style in this field. In some healthcare settings, nursing tasks are highly standardized, and work content and responsibilities are clearly defined. Therefore, older nurses may rely more on established workflows and are less likely to propose innovative ideas or changes to the existing practices [27]. Moreover, the relatively small sample size of nurses aged 50 years and above in this study (only 1.4%) may have affected the stability and representativeness of the results. The small sample size may have obscured potential differences in voice behavior between older and younger nurses. Thus, sample size limitation could be a key factor contributing to the lack of significant findings. Future studies could expand the sample size to further explore differences in voice behavior across age groups, particularly in different cultural and work environments.

#### 4.2.2. Marital Status

The results of this study indicate that unmarried individuals had the lowest scores for voice behavior. This phenomenon may be related to differences in social roles, family responsibilities, and work-related stress among the unmarried group. Unmarried employees typically do not have family burdens, which may allow them to focus more on personal career development and emotional needs, leading to less involvement in organizational voice behaviors. Moreover, unmarried employees tend to have a more limited social support system, which may affect their ability to express opinions and provide feedback within the organization [27]. This study also found that married and divorced individuals showed different behaviors in terms of prohibitive voice. Divorced nurses were more likely to engage in prohibitive voice behavior compared to married nurses (i.e., actively pointing out potential negative factors or problems in the workplace). Prohibitive voice refers to employees pointing out and attempting to eliminate negative factors that may affect work efficiency or cause harm, even if such behavior may lead to discomfort or dissatisfaction among others [24]. This result may be related to the emotional and psychological pressures experienced by divorced individuals. Divorce can impact an individual's social identity, leading to more direct and intense expressions of dissatisfaction at work. As a result, they may choose a more proactive approach to voice, such as using prohibitive voice to express their disappointment or anger regarding workplace issues [26]. Furthermore, the rising divorce rate in China reflects changes in public attitudes toward marriage and family. According to data from the China Economic Information Network (https://www.cei.cn/), the national divorce rate has increased from 0.96% in 2000 to 3.09% in 2020. This shift suggests that divorced individuals may have different attitudes toward social norms and workplace behavior after experiencing marital dissolution. These changes could influence their attitudes and behaviors at work, including their tendency to use prohibitive voice to express emotions and views when dissatisfied with work [28]. For married nurses, however, they generally face higher family responsibilities and may prioritize work stability, avoiding unnecessary conflicts or risks at work. As a result, they may be more cautious in expressing negative opinions [25]. Married nurses are more likely to rely on stable work processes and prefer maintaining the status quo rather than suggesting changes or innovations.

Therefore, the influence of marital status on voice behavior is not only related to changes in social roles but also closely linked to emotional support, family responsibilities, and psychological states. Divorced individuals may be more inclined to express dissatisfaction through prohibitive voice, while married employees tend to focus on work stability and team harmony, thus displaying more caution. Future research should further explore the differences in voice behaviors across different marital status groups, particularly in different cultural and work environments.

#### 4.2.3. Number of Children

This study found that nurses with more children are more likely to engage in voice behavior, and the number of children positively predicts voice behavior. This result may be closely related to family responsibilities, social support, and employee motivation. Research indicates that employees with more children may focus more on work efficiency and improvement due to the increased family responsibilities, aiming to ensure family stability [27]. Additionally, employees with more children typically have a stronger social support system, which may enhance their confidence in expressing opinions at work [29]. While heavier family responsibilities may increase work-related stress, some studies suggest that employees may reduce their voice behavior due to the burdens of family obligations [28]. However, this study shows that the number of children is positively correlated with voice behavior, indicating that family responsibilities do not significantly inhibit voice behavior. Instead, the focus on work stability may encourage employees to participate more actively in work improvement. Therefore, it can be inferred that the impact of the number of children on voice behavior is multifaceted, involving both the pressures of family responsibility and the positive influence of family support. Future research could further explore the role of family factors in different work environments, particularly how family responsibilities influence employees' voice behavior.

#### 4.2.4. Job Title and Position

This study found no significant difference in the voice behavior between nurses and junior nurses, which may be attributed to the relatively limited work experience of both groups. Research suggests that employees with less experience, especially those with lower titles, are less likely to engage in voice behavior, particularly when faced with potential conflicts or complex issues, as their confidence and job capabilities are not fully developed [24]. Employees with less experience are typically more inclined to avoid taking on too much responsibility, especially when it involves offering criticism or negative feedback. The study also found that nurses with higher titles are more likely to engage in prohibitive voice behavior. This phenomenon may be related to the fact that nurses with higher titles have accumulated more work experience and hold greater job responsibilities. Research indicates that more experienced and higher ranking employees are more willing to provide negative feedback or critical opinions, especially regarding work quality and patient safety [25]. Senior nurses usually possess strong professional knowledge and judgment, which gives them more confidence in identifying potential risks and problems, although this behavior may lead to conflicts with superiors or colleagues [29].

Regarding job position, the study found that nurses in higher positions are more likely to engage in voice behavior, and regression analysis revealed that job position is a negative predictor of voice behavior, particularly in terms of prohibitive voice. High-ranking employees typically assume more managerial responsibilities and wield greater influence within the organization. As such, their voice behavior is often more focused on organizational-level improvements or feedback on issues, especially at higher management and decision-making levels [28]. However, as position level increases, employees' voice behavior may become more cautious, particularly when offering critical opinions, as they may be concerned about affecting their relationships with colleagues or superiors [25]. Therefore, while higher ranking employees may demonstrate more voice behavior in some areas, their contributions are often more negative, especially when addressing potential organizational risks. Future research could further explore the multidimensional impact of job title and position on voice behavior, particularly how to balance positive and negative voice behaviors in different levels of work environments.

#### 4.2.5. Years of Experience

This study found no significant difference in voice behavior scores between nurses with 6–10 years and 11–15 years of experience, with scores of 41.92 ± 6.56 and 42.61 ± 6.31, respectively, and no statistically significant differences were found in multiple comparisons. This phenomenon may be closely related to the current training program for neonatal nurses at the hospital. Depending on their entry-level education and employment plans, nurses with 6–10 years and 11–15 years of experience may have differences in job titles, such as junior nurse and charge nurse. Moreover, nurses in both groups mainly undertake clinical foundational work and specialized procedures, with relatively minor differences in job content. As a result, no significant differences were observed in their voice behavior. Since the responsibilities of these nurses are similar and they face comparable work pressures and challenges, their voice behavior tends to be relatively consistent, leading to no noticeable difference in voice behavior between nurses with different years of experience.

## 5. Conclusions

The voice behavior of NICU nurses is influenced by multiple factors, including demographic characteristics such as age, marital status, number of children, job title, position, and years of experience. However, these factors interact in complex ways, and nursing managers must consider both individual characteristics and the broader work environment when developing management strategies. In particular, it is essential to address prohibitive voice behavior among senior nurses, as fostering their active participation can significantly enhance the quality of care. Furthermore, leadership styles and organizational culture, as well as factors such as team dynamics and work environment, should be carefully considered to create a more inclusive and supportive environment.

To achieve this, healthcare organizations can implement strategies that promote open communication, psychological safety, and trust within nursing teams. These efforts will help ensure that nurses feel empowered to voice their opinions and contribute to decision-making processes, ultimately improving patient outcomes. Additionally, nursing managers should be mindful of emerging trends, such as the increasing diversity of the nursing workforce and changes in healthcare delivery models, as these factors may further influence voice behavior and shape future nursing management practices. By leveraging these insights, healthcare organizations can create a supportive environment that nurtures effective communication, enhances collaboration, and contributes to continuous improvement in the NICU.

## 6. Implications for Nursing Management

The findings of this study have important implications for nursing management. Nursing managers can adopt the following strategies to enhance nurses' participation in decision-making processes and, consequently, improve the quality of care:

1. Creating a supportive and open work environment: The work environment is a key factor influencing voice behavior. A supportive and open environment can effectively encourage nurses to express their opinions, especially when supported by inclusive leadership. Inclusive leadership, by fostering a culture of fairness and respect, can significantly promote employee voice behavior [30]. Regardless of nurses' marital status or number of children, all nurses should be encouraged to voice their opinions. Nursing managers should prioritize creating a psychologically safe environment where all nurses feel free to express their views without the fear of retaliation. Psychological safety plays a crucial role in promoting voice behavior, as nurses are more likely to speak up when they believe their opinions will be valued and will not lead to negative consequences [25]. Managers can ensure that all nurses' voice behaviors are given attention and respect through training and supportive leadership styles, thereby enhancing team communication and collaboration.
2. Addressing prohibitive voice behavior: Experienced nurses, especially those with senior titles, are often more inclined to engage in prohibitive voice behavior, such as offering criticism or negative feedback, particularly regarding critical issues such as patient safety and work efficiency. While such feedback is crucial for identifying problems and improving practices, it can also lead to internal conflicts or create a negative atmosphere within the team, especially if not properly managed. Therefore, nursing managers need to adopt appropriate strategies to balance the positive impact of this behavior with its potential negative effects. Nursing managers should focus on guiding this prohibitive voice behavior, helping it transform into more constructive promotive voice behavior, thereby driving work improvements and enhancing team collaborations [31]. Senior nurses typically possess stronger professional judgment and confidence, enabling them to identify issues and provide critical feedback. However, nursing managers should offer training to help them express their opinions in a more collaborative and positive manner, ensuring that their feedback contributes to problem-solving while maintaining harmony and efficiency within the team.
3. Improving leadership styles and communication channels: Leadership style is a key factor influencing voice behavior. Paradoxical leadership refers to leaders who can motivate employees to speak up by balancing opposing demands. This leadership style has been shown to promote voice behavior [32, 33]. Leaders with paradoxical thinking are able to drive organizational goals while encouraging employees to express their opinions, thereby fostering organizational improvement. Hospital leadership should focus on cultivating paradoxical thinking and communication skills among nursing managers to help them enhance nurses' involvement while balancing management needs with employee expression. Additionally, nursing managers should provide nurses with clear and accessible communication channels to ensure that their suggestions are heard and valued. When nurses feel that their feedback is taken seriously, they are more likely to engage in discussions and propose innovative solutions [25].
4. Developing targeted and tiered management strategies: Work experience has a significant impact on voice behavior. Nurses with less experience tend to participate less in voice behavior due to a lack of confidence or fear of conflict [31]. Nursing managers can develop targeted strategies based on nurses' work experience and job levels. For newly hired or less experienced nurses, more support and guidance should be provided to help them build confidence and gradually encourage their participation in voicing their opinions in the workplace. For more experienced nurses, managers can encourage them to actively engage in the decision-making process and provide constructive feedback through leadership development programs, fostering work improvement. Additionally, nursing managers should adopt tiered management strategies, grouping nurses based on their work experience and job levels, rather than solely relying on personal characteristics such as marital status or number of children. For example, experienced nurses can serve as team leaders, responsible for guiding and leading other nurses to ensure team collaboration and efficiency. Newly hired nurses can be better integrated into the team through pairing and mentoring, enhancing their sense of involvement and confidence, which will help them more actively voice their opinions in daily work.
5. Promoting team cooperation and collaboration: Effective voice behavior is often influenced by team cooperation. Research shows that trust and collaboration among team members significantly enhance voice behavior [32]. Nursing managers should focus on strengthening teamwork, fostering mutual respect and trust among team members. Regular team meetings and discussions, where nurses can freely express their opinions in a nonjudgmental environment, are keys to promoting collaboration and continuous improvement.
6. Enhancing organizational support and incentive mechanisms: Organizational support is crucial for nurses' voice behavior, particularly in areas related to patient safety and work efficiency. Nurses who feel supported by the organization are more willing to offer suggestions and feedback [24]. Nursing managers should enhance organizational support by providing necessary resources, systematic training, and positive feedback, encouraging nurses to contribute improvement suggestions and innovative ideas in their work. At the same time, managers can design incentive mechanisms to encourage all nurses to voice their opinions and participate in work improvement, rather than relying solely on promotions as a reward. By establishing reward systems (such as recognizing nurses who actively voice their suggestions or rewarding innovative ideas), nurses can be motivated to take a more proactive role in team building and decision-making, thereby promoting collaboration and improving the quality of care. Furthermore, the study highlights that organizational trust not only directly influences nurses' voice behavior but also indirectly promotes it by enhancing career resilience [34]. Therefore, enhancing organizational support and trust will more effectively promote nurses' voice behavior, ultimately contributing to improved work environments and patient care quality.

## 7. Limitations and Future Directions

This study relied on self-reported data, which may introduce recall bias and social desirability bias. Participants may have been inclined to provide answers that align with social expectations or what they perceive as more acceptable, especially in a hierarchical work environment such as healthcare. This could potentially lead to an overestimation of voice behavior. Although measures were taken to ensure anonymity and voluntary participation, completely eliminating self-report bias remains a challenge. Future research could consider incorporating other data collection methods, such as qualitative interviews or objective indicators, to enhance the accuracy and reliability of the findings.

Despite a coverage rate of 85.71%, this study did not fully cover all prefecture-level cities and autonomous regions in Sichuan Province. Future research could further expand the sample size and compare regional differences to provide more precise guidance for regional interventions.

In this study, male nurses accounted for only 0.07% (3/422) of the sample. To reduce statistical errors, gender was not included in the analysis. As the proportion of male nurses gradually increases in the healthcare industry, gender differences may have a varying impact on voice behavior. Future studies should consider expanding the sample size, particularly in terms of gender diversity, to more comprehensively assess the potential impact of gender differences on variables such as voice behavior.

Furthermore, the majority of the sample in this study was Han Chinese (96.0%), with a small proportion of ethnic minorities (such as Tibetan and Yi). According to the ethnic composition of China, Han Chinese account for about 91.1% of the total population, while ethnic minorities comprise about 8.9%. This ethnic homogeneity is more common in certain regions and industries and may impact the understanding of voice behavior among nurses from different ethnic backgrounds. Future research could consider expanding the sample of ethnic minority nurses and explore the potential impact of different cultural backgrounds on voice behavior, thereby improving the generalizability and reliability of the results. Furthermore, cross-cultural and gender-difference studies could provide a more comprehensive understanding of how cultural factors influence nurses' voice behavior.

Additionally, the existing studies have shown that a high-trust organizational environment can significantly enhance employees' willingness to voice, and career resilience helps nurses maintain a positive attitude when facing professional challenges, further strengthening their voice behavior. Therefore, future research could focus on exploring the impact of factors such as organizational trust and career resilience on nurses' voice behavior. A longitudinal research design could be employed, involving a long-term tracking of the same group of nurses, to analyze how these factors continuously influence the evolution of voice behavior at different career stages (such as early career, mid-career, and preretirement). This design would help reveal the temporal dynamics of voice behavior, providing more targeted and forward-looking recommendations for nursing management, ultimately contributing to the improvement of healthcare quality.

## Figures and Tables

**Table 1:** Basic characteristics of study participants (*N* = 422).

Demographic variable	Category	Frequency	Percentage (100%)
Gender	Female	419	99.3
	Male	3	0.7

Age	< 30 years	135	32.0
	30∼39 years	223	52.8
	40∼49 years	58	13.8
	≥ 50 years	6	1.4

Ethnicity	Han	405	96.0
	Tibetan	13	3.1
	Yi	4	0.9

Only child status	Yes, only child	132	31.3
	No, one sibling	220	52.1
	No, two siblings	50	11.9
	No, three or more siblings	20	4.7

Marital status	Unmarried	92	21.8
	Married	319	75.6
	Divorced	11	2.6

Number of children	No children	132	31.3
	One child	197	46.7
	Two children	93	22.0

Education level	Master's degree or above	11	2.6
	Bachelor's degree	346	82.0
	Associate degree	65	15.4

**Table 2:** Other work-related information (*N* = 422).

Variable	Category	Frequency	Percentage (100%)
Type of hospital	General hospital	321	76.1
	Specialized hospital	101	23.9

Job title	Chief nurse	3	0.7
	Associate chief nurse	19	4.5
	Supervisor nurse	175	41.5
	Senior nurse	193	45.7
	Nurse	32	7.6

Position	Head/Deputy head nurse	25	5.9
	Key staff/Team leader	62	14.7
	Frontline nurse	335	79.4

Years of experience in NICU	≤ 5 years	155	36.7
	6∼10 years	129	30.6
	11∼15 years	83	19.7
	> 15 years	55	13.0

**Table 3:** Voice behavior scale (*N* = 422).

Dimension	Item	Cronbach's α if item deleted	Cronbach's *α*	
Promotive voice	A1	0.947	0.950	0.951
	A2	0.945		
	A3	0.943		
	A4	0.943		
	A5	0.943		
Prohibitive voice	A6	0.945	0.911	
	A7	0.946		
	A8	0.950		
	A9	0.948		
	A10	0.945		

**Table 4:** Differences in voice behavior among different study participants.

Variable	Category	Total voice behavior score			Promotive voice			Prohibitive voice		
		*M* ± SD	*t/F*	*p*	*M* ± SD	*t/F*	*p*	*M* ± SD	*t/F*	*p*
Age	< 30 years	38.14 ± 7.44	22.342⁣^∗^	< 0.001	19.59 ± 3.89	18.447⁣^∗^	< 0.001	18.55 ± 4.11	20.575⁣^∗^	< 0.001
	30∼39 years	42.32 ± 6.37			21.70 ± 3.45			20.62 ± 3.40		
	40∼49 years	46.14 ± 5.28			23.38 ± 2.66			22.76 ± 2.90		
	≥ 50 years	43.33 ± 8.19			21.83 ± 3.72			21.50 ± 4.18		

Ethnicity	Han	41.51 ± 7.11	0.447	0.640	21.27 ± 3.70	6.745⁣^∗^	0.318	20.24 ± 3.84	0.364	0.695
	Tibetan	41.00 ± 8.12			20.38 ± 4.68			20.62 ± 4.05		
	Yi	44.75 ± 4.11			23.00 ± 2.16			21.75 ± 2.36		

Only child status	Yes, only child	41.31 ± 6.98	1.423	0.236	21.21 ± 3.68	1.004	0.391	20.10 ± 3.85	1.567	0.197
	No, one sibling	41.79 ± 7.06			21.37 ± 3.64			20.42 ± 3.80		
	No, two siblings	42.10 ± 6.82			21.44 ± 3.78			20.66 ± 3.44		
	No, three or more siblings	38.55 ± 8.89			19.90 ± 4.69			18.65 ± 4.75		

Marital status	Unmarried	39.18 ± 7.31	6.853	0.001	20.00 ± 3.85	7.484	0.001	19.18 ± 3.90	4.851	0.008
	Married	42.12 ± 6.94			21.57 ± 3.62			20.55 ± 3.78		
	Divorced	43.82 ± 6.62			22.73 ± 3.47			21.09 ± 3.42		

Number of children	No children	38.57 ± 7.60	20.607	< 0.001	19.69 ± 3.92	21.362	< 0.001	18.88 ± 4.17	15.172	< 0.001
	One child	42.26 ± 6.61			21.65 ± 3.44			20.61 ± 3.55		
	Two children	44.16 ± 5.95			22.67 ± 3.23			21.49 ± 3.32		

Education level	Master's degree or above	42.18 ± 9.10	0.231	0.794	21.73 ± 4.50	0.170	0.844	20.45 ± 4.93	0.281	0.755
	Bachelor's degree	41.60 ± 7.06			21.28 ± 3.70			20.32 ± 3.80		
	Associate degree	41.02 ± 7.13			21.08 ± 3.74			19.94 ± 3.86		

⁣^∗^The Welch test used due to unequal variances.

**Table 5:** Differences in voice behavior among other work-related factors.

Variable	Category	Total voice behavior score			Promotive voice			Prohibitive voice		
		*M* ± SD	*t/F*	*p*	*M* ± SD	*t/F*	*p*	*M* ± SD	*t/F*	*p*
Type of hospital	General hospital	41.63 ± 7.12	0.543	0.587	21.34 ± 3.72	0.745	0.456	20.29 ± 3.84	0.284	0.776
	Specialized hospital	41.19 ± 7.10			21.02 ± 3.74			20.17 ± 3.82		

Job title	Chief nurse	49.33 ± 0.58	36.042⁣^∗^	< 0.001	25.00 ± 0.00	—	—	24.33 ± 0.58	20.401⁣^∗^	< 0.001
	Associate chief nurse	47.05 ± 4.58			23.74 ± 2.60	63.311⁣^∗^	< 0.001	23.32 ± 2.24		
	Supervisor nurse	43.42 ± 6.18			22.22 ± 3.23			21.21 ± 3.33		
	Senior nurse	39.66 ± 7.28			20.41 ± 3.84			19.24 ± 3.99		
	Nurse	38.38 ± 7.41			19.31 ± 3.91			19.06 ± 3.94		

Position	Head/Deputy head nurse	48.92 ± 2.60	82.626⁣^∗^	< 0.001	24.52 ± 1.36	82.997⁣^∗^	< 0.001	24.40 ± 1.32	85.752⁣^∗^	< 0.001
	Key staff/Team leader	43.6 ± 5.59			22.48 ± 3.01			21.11 ± 3.05		
	Frontline nurse	40.59 ± 7.20			20.79 ± 3.79			19.80 ± 3.88		

Years of experience	≤ 5 years	39.07 ± 7.67	19.893⁣^∗^	< 0.001	20.11 ± 4.07	191.500⁣^∗^	< 0.001	18.96 ± 4.17	191.565⁣^∗^	< 0.001
	6∼10 years	41.92 ± 6.56			21.29 ± 3.48			20.63 ± 3.51		
	11∼15 years	42.61 ± 6.31			21.98 ± 3.25			20.64 ± 3.44		
	> 15 years	45.85 ± 5.06			23.35 ± 2.66			22.51 ± 2.68		

*Note:* Variance for Chief Nurses in promotive voice behavior is 0; thus, data for this category were excluded for analysis.

⁣^∗^Welch test used due to unequal variances.

**Table 6:** Multiple comparison results for voice behavior in relation to general characteristics of the study subjects.

Variable	Multiple comparison method	(I) Age group (years)	(J) Age group (years)	Score of voice behavior				Promotive voice				Prohibitive voice			
				SE	*p*	95% CI		SE	*p*	95% CI		SE	*p*	95% CI	
Age	Tamhane's T2^b^	≤ 30	31∼40	0.722⁣^∗^	0.000	−5.600	−2.760	0.407⁣^∗^	0.000	−3.190	−1.030	0.420⁣^∗^	0.000	−3.190	−0.960
			41∼50	1.040⁣^∗^	0.000	−10.040	−5.950	0.484⁣^∗^	0.000	−5.080	−2.500	0.520⁣^∗^	0.000	−5.600	−2.820
			≥ 51	2.764	0.061	−10.630	0.240	1.675	0.799	−8.990	4.500	1.744	0.614	−9.970	4.060
		31∼40	41∼50	0.977⁣^∗^	0.000	−5.730	−1.900	0.419⁣^∗^	0.001	−2.800	−0.550	0.444⁣^∗^	0.000	−3.330	−0.950
			≥ 51	2.741	0.713	−6.400	4.380	1.658	1.000	−6.950	6.690	1.723	0.997	−7.980	6.220
		41∼50	≥ 51	2.841	0.324	−2.780	8.390	1.678	0.951	−5.190	8.280	1.750	0.985	−5.730	8.250

Marital status	LSD	Unmarried	Married	0.830⁣^∗^	0.000	−4.570	−1.300	0.434⁣^∗^	0.000	−2.430	−0.720	0.449⁣^∗^	0.003	−2.240	−0.480
			Divorced	2.238⁣^∗^	0.016	−9.030	−0.230	1.169⁣^∗^	0.010	−5.030	0.430	1.212⁣^∗^	0.016	−4.290	−0.480
		Married	Divorced	2.151	0.430	−5.930	2.530	1.124	0.305	−3.360	1.060	1.165⁣^∗^	0.040	−2.830	−1.740

Number of children	LSD	No children	1 child	0.765⁣^∗^	0.000	−5.190	−2.190	0.400⁣^∗^	0.000	−2.750	−1.170	0.417⁣^∗^	0.000	−2.550	−0.910
			2 children	0.921⁣^∗^	0.000	−7.400	−3.780	0.481⁣^∗^	0.000	−3.920	−2.030	0.502⁣^∗^	0.000	−3.600	−1.630
		1 children	2 children	0.856	0.017	−3.580	−0.220	0.447	0.013	−1.900	−0.140	0.467	0.015	−1.800	−0.230

⁣^∗^The significance level for the difference between the means was 0.05.

^b^Indicates unequal variance, Tamhane's T2 test applied.

## Data Availability

The datasets generated and/or analyzed during the current study are not publicly available due to privacy concerns for the research participants. However, they are available from the corresponding author upon reasonable request.
